# Dynamics of multiple insecticide resistance in the malaria vector *Anopheles gambiae *in a rice growing area in South-Western Burkina Faso

**DOI:** 10.1186/1475-2875-7-188

**Published:** 2008-09-25

**Authors:** Kounbobr Roch Dabiré, Abdoulaye Diabaté, Luc Djogbenou, Ali Ouari, Raphaël N'Guessan, Jean-Bosco Ouédraogo, Jean-Marc Hougard, Fabrice Chandre, Thierry Baldet

**Affiliations:** 1 Institut de Recherche en Science de la Santé (IRSS)/Centre Muraz, BP 390, Bobo-Dioulasso, Burkina Faso; 2 Laboratory of Malaria and Vector Research/NIAID/NIH, Rockville, Washington, USA; 3 Institut de Recherche pour le Développement (IRD), Cotonou, Bénin; 4 Centre de Recherche Entomologique de Cotonou (CREC), Cotonou, Bénin; 5 Cirad, UPR15, Campus international de Baillarguet, Montpellier, France

## Abstract

**Background:**

Insecticide resistance of the main malaria vector, *Anopheles gambiae*, has been reported in south-western Burkina Faso, West Africa. Cross-resistance to DDT and pyrethroids was conferred by alterations at site of action in the sodium channel, the Leu-Phe *kdr *mutation; resistance to organophosphates and carbamates resulted from a single point mutation in the oxyanion hole of the acetylcholinesterase enzyme designed as *ace-1*^*R*^.

**Methods:**

An entomological survey was carried out during the rainy season of 2005 at Vallée du Kou, a rice growing area in south-western Burkina Faso. At the Vallée du Kou, both insecticide resistance mechanisms have been previously described in the M and S molecular forms of *An. gambiae*. This survey aimed i) to update the temporal dynamics and the circumsporozoite infection rate of the two molecular forms M and S of *An. gambiae *ii) to update the frequency of the Leu-Phe *kdr *mutation within these forms and finally iii) to investigate the occurrence of the *ace-1*^*R *^mutation.

Mosquitoes collected by indoor residual collection and by human landing catches were counted and morphologically identified. Species and molecular forms of *An. gambiae*, *ace-1*^*R *^and Leu-Phe *kdr *mutations were determined using PCR techniques. The presence of the circumsporozoite protein of *Plasmodium falciparum *was determined using ELISA.

**Results:**

*Anopheles gambiae *populations were dominated by the M form. However the S form occurred in relative important proportion towards the end of the rainy season with a maximum peak in October at 51%. Sporozoite rates were similar in both forms. The frequency of the Leu-Phe *kdr *mutation in the S form reached a fixation level while it is still spreading in the M form. Furthermore, the *ace*-*1*^*R *^mutation prevailed predominately in the S form and has just started spreading in the M form. The two mutations occurred concomitantly both in M and S populations.

**Conclusion:**

These results showed that the Vallée du Kou, a rice growing area formerly occupied mainly by M susceptible populations, is progressively colonized by S resistant populations living in sympatry with the former. As a result, the distribution pattern of insecticide resistance mutations shows the occurrence of both resistance mechanisms concomitantly in the same populations. The impact of multiple resistance mechanisms in M and S populations of *An. gambiae *on vector control measures against malaria transmission, such as insecticide-treated nets (ITNs) and indoor residual spraying (IRS), in this area is discussed.

## Background

Malaria transmission in tropical Africa is mainly dominated by *Anopheles gambiae *complex, including *An. gambiae *s.s., the most anthropophilic vector transmitting malaria in sub-Saharan Africa [1]. Formerly considered as a single species, it began early to accumulate genetic heterogeneity. In the 1980s, cytogenetic studies based on chromosomal inversion arrangements found five incipient chromosomal forms. The suspected role of these inversions was to restrict gene flow among the forms and to provide adaptation to different ecological settings [2,3]. In Burkina Faso, *Mopti *and *Savanna *chromosomal forms dominated *An. gambiae *population structure [4]. These chromosomal forms appeared more or less genetically isolated in the field, presumably through prezygotic barriers since viable and fertile hybrids have been obtained in the laboratory [5-7]. However, cytogenetic analysis is not a precise way to evaluate the degree of hybridization between forms because of the presence of cryptic 'heterokaryotypes'. Recent studies based on molecular markers such as X-linked ribosomal DNA suggested the existence of only two entities within *An. gambiae*, referred to as M and S molecular forms [8]. So far, in Burkina Faso and Mali, in savannah environments of West Africa, *Mopti *and *Savanna *chromosomal forms coincide respectively with M and S molecular forms [4]. As a main malaria major vector with high level of polymorphism, *An. gambiae *has been a subject of many investigations in West Africa, such as bio-ecology and insecticide resistance studies [9-12].

In Burkina Faso, a study carried out in the mid-1980s in Vallée du Kou [13], showed that the *Mopti *chromosomal form was dominating. Molecular-based identification of *An. gambiae *s.s. populations conducted in this area in 1999 and 2000 confirmed that the M molecular form predominated throughout the year [11,12,14], although with some temporal variations.

The S molecular form occurred in low frequency until the end of the rainy season (October/November), when it peaked around 30% [12], as found previously by Robert *et al *[13]. A similar pattern was found in the same environment in Mali [7,9]. More recently, in 2004 the S form was observed in Vallée du Kou towards the end of the rainy season at a frequency of 50% [15]. This study gave no results regarding the frequencies of the two forms throughout the malaria transmission season.

In West Africa, the main mechanism involved in pyrethroid-resistance in *An. gambiae *is caused by target site insensitivity through a knockdown resistance (*kdr*)-like mutation caused by a single point mutation (Leu-Phe) in the *para*-sodium channel gene [16]. Preliminary surveys done in Vallée du Kou in 1999 indicated that the Leu-Phe *kdr *mutation has been found almost only in the S form at high allelic frequency (0.95) compared to just 0.006 in the M form [12]. However, the spread of the mutation in the M population seemed an ongoing process in Vallée du Kou as it increased to a frequency of 0.02 in 2000. Nowadays, the population structure of *An*.*gambiae *and their pyrethroid resistance status are probably modified with the changing in agricultural practices needing intensive use of insecticides (cotton and vegetable cropping) and also the increasing of man made breeding sites as puddles throughout the village.

Furthermore, it has been recently noted in *An. gambiae *from the same area the occurrence of a single point mutation (glycine to serine at position 119) in the oxyanion hole of the acetylcholinesterase enzyme [17]. This mutation named *ace-1*^*R *^mutation was associated with insensitivity of *An. gambiae *to organophosphates and carbamates [18].

The objective of the present study was to gather recent information on the dynamics of the two molecular forms of *An. gambiae *throughout the malaria transmission season in this area with particular attention to resistance mechanisms. This information is crucial for a proper evaluation of new insecticides or vector control tools expected to be involved in malaria control and resistance management.

## Materials and methods

### Study site

**Figure 1:**
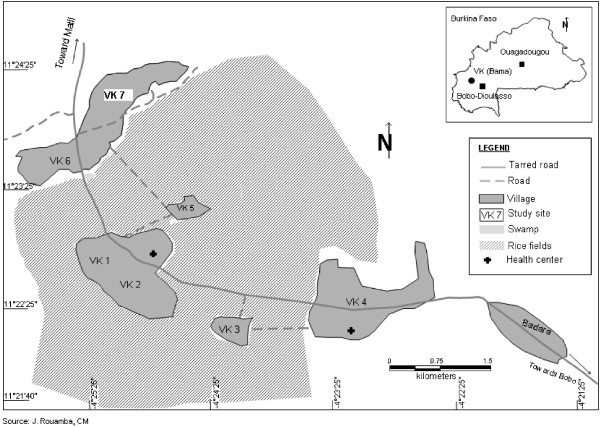
Location of the study site.

Vallée du Kou (4°25' W, 11°24' N) is a rice-growing valley covering 1,200 ha and comprising seven villages, with a total of 4,470 habitants surrounded by humid savannah. The rainy season extends from June to October and the dry season from November to May. The Kou River is a permanent source of irrigation water and there are two rice crops per year from July to November and from January to May. Few insecticides are used on rice, but huge amounts of insecticides are used extensively in cotton fields' located on the outside of rice fields. During the last two years, some producers started to grow vegetables involving intensive use of insecticides. Mosquito collection had been carried out in the village numbered as seven (VK7), which is located at the end of the rice fields (Figure 1). VK7 has about 600 inhabitants, mainly farmers. Sheep, goats, pigs, and a few cows are also present. Cotton and maize fields surrounded this village.

The irrigation system and rice fields provide year-round mosquito breeding. Additional breeding sites are created by rains in the depressions and ponds. High densities of *An. gambiae *(up to 200 bites/person/night) are recorded during the rainy season [14]. The two molecular forms M and S of *An. gambiae *occur in sympatry notably at the end of the rainy season [12,15]. The *kdr*-based mechanism conferring resistance to pyrethroids and DDT and the *ace-1*^*R *^mutation conferring resistance to organophosphates and carbamates predominate in the S form [11,12,17].

### Mosquito collections

Anopheline mosquitoes were sampled during the rainy season from July to November 2005 mainly by indoor manual collection for resting mosquitoes and, secondarily by human landing catches.

Indoor resting mosquitoes were collected regularly in four houses in the village early in the morning by manual aspirators four times a month, on four consecutive days. These indoor collections carried out monthly from July to November were used to establish the temporal dynamics of the two molecular forms of *An. gambiae *and their resistance status by PCR.

Human landing catches were ensured only in August and in October to evaluate the sporozoite infection rate of each molecular form. These two periods correspond to the peak of the M form in August and of the S form in October. Catches were carried out between 20.00 and 06.00, during two consecutive nights for each period inside and just outside of four houses of the village. They were performed by informed volunteers who were provided free and rapid treatment when suspected clinical signs of malaria according to World Health Organization (WHO)-recommended regimen on the basis of fever and detectable *Plasmodium falciparum *parasitemia.

### Laboratory processing of mosquitoes

Anophelines were sorted and assigned to species based on morphological characters using standard identification keys [19]. Females referring to *An. gambiae *complex were processed by PCR concomitantly for identification of species and molecular forms of *An. gambiae *[20,8]. Detection of Leu-Phe *kdr *and *ace-1*^*R *^mutations were performed on indoor resting specimens by PCR from genomic DNA following Martinez-Torres *et al *[21] and Weill *et al *[22], respectively.

The head-thoraces of anopheline females issued from human landing catches were tested for the presence of the circumsporozoite protein (CSP) of *P. falciparum*, the major malarial parasite occurring in the study area, by enzyme-linked immunosorbent analysis (ELISA) according to Wirtz *et al *[23]. Samples of August were used for ELISA process because this month corresponded to the peak of the M form whereas no or few individuals of S form were found in indoor collections.

### Data analysis

The sporozoite rate was defined as the proportion of mosquitoes found positive for *P. falciparum *CS protein. The entomological inoculation (EIR) was calculated as the product of HBR and the sporozoite rate of mosquitoes caught on landing collections.

## Results

### Dynamics of the M and S molecular forms

**Figure 2:**
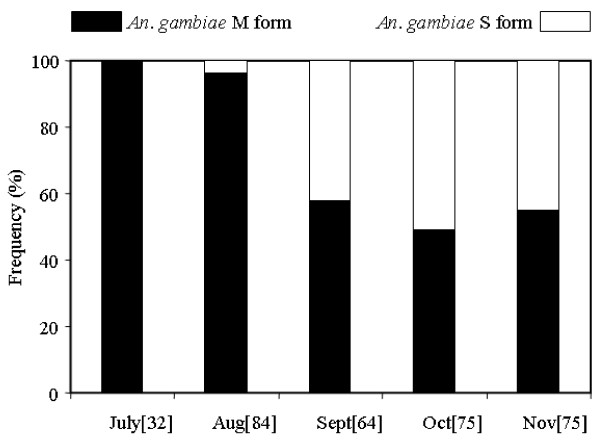
Monthly variation of the relative prevalence of the molecular M and S forms of *Anopheles gambiae *s.s.

Overall, 330 mosquitoes were analysed for identification of species and molecular forms of *An. gambiae *(Figure 2). *Anopheles arabiensis *was absent. Both M and S molecular forms occurred. The overall frequency of the molecular M form was higher than that of the S form reaching respectively 69% *versus *31%, but the relative prevalence of these forms throughout the collecting period showed some monthly varying frequencies. During the first part of the rainy season (July and August), the M form predominated, whereas the two forms are found in similar proportion during the second part of the rainy season (from September to November). Indeed in July all mosquitoes analysed (n = 32) were only of the M form. The S form started appearing toward the end of August at low frequency of 4% (3/84) reaching a maximum peak of 51% (38/75) in October equalling the frequency of the M form.

### Sporozoite rate

The sporozoite rate was determined in *An. gambiae *females issued from indoor human landing catches carried out in two sampling sets: August and October 2005 (Table 1). Because in August no specimens of the S form were found, sporozoite rate was determined only by the M form reaching 1.19%.

**Table 1:** Circumsporozoite infection rate for *Plasmodium falciparum *determined by ELISA in specimens issued from indoor/outdoor human landing catches carried out in VK7 in August and October 2005

	August		October	
	
Molecular form of *An. gambiae*	Nb tested	CSPR	Nb tested	CSPR
M	84 (1)	1.19 [0.03–6.46]	54 (1)	1.85 [0.05–9.89]
S	0	-	11 (1)	9.09 [0.23–41.28]

() number of CSP-positive mosquitoes

[]: 95% confidence interval.

In October, the M and S forms were collected in equal numbers. Results yielded an average sporozoite rate of 3.08% with no significant difference between the two forms (Fisher's Exact test, *P *= 0.33).

### Distribution of the *kdr *mutation

An average of 49 mosquitoes issued from indoor resting fauna were analysed monthly for the Leu-Phe *kdr *mutation (Table 2). The *kdr *mutation occurred in both M and S forms with varying allelic frequencies in the M form. *Kdr *frequency was higher in the S form irrespective of the month (χ^2 ^= 6.14, df = 2, *P *< 0.02) reaching 0.93 in mean and ranged from 0.50 to 0.98 throughout its occurrence period. None homozygous sensible individual was identified from the S form. The mean frequency of the *kdr *mutation in the form M was 0.096 and ranged from 0 to 0.23 throughout the five months. Only 8 mosquitoes from the M form were identified in September and November as homozygous RR for this mutation vs. 15 hybrids RS overall among 161 specimens analysed. In the S form the homozygous resistant predominated irrespective of the month contributing thus to achieve the fixation process of this allele in the S population.

**Table 2:** Monthly variation in the frequency of the *kdr *mutation and the relative prevalence of the two molecular forms of *Anopheles gambiae *ss in VK7 from July to November 2005

	S form^a^	*Kdr*^b^	F(R)^c^	M form^a^	*Kdr*^b^	F(R)^c^
	
July	0-	-	-	32 100%	31SS + 1ND	0
August	3 11.5%	3RS	0.50	23 88.5%	3RS+20SS	0.065
September	23 39.0%	22RR+1RS	0.98	36 61.0%	3RR+3RS+30SS	0.125
October	29 46.8%	25RR+3RS+1SS	0.91	33 53.2%	2RS+31SS	0.03
November	28 43.1%	24RR+3RS	0.94	37 56.9%	5RR+7RS+25SS	0.23
Total	83 34.0%	71RR+10RS +2ND	0.93	161 66.0%	8RR+15RS+137SS +1ND	0.097

^a ^Number of mosquitoes analysed, relative prevalence of molecular forms of *An. gambiae *(in percentage)

^b ^Genotype of the *kdr *mutation. ^c^ Allelic frequency of the *kdr *mutation in mosquitoes analysed

ND: undetermined

[the global prevalence of the M form is more higher than that of the S form, χ^2 ^= 84.92, df = 1, P < 0.001].

### Distribution of the *ace-1^*R *^*mutation

58 mosquitoes issued from indoor resting fauna were analysed for the *ace-1*^*R *^mutation from September and October 2005, corresponding to 29 specimens per month (Table 3). The *ace-1*^*R *^mutation was detected at low frequency in the M form with only one heterozygous RS per month corresponding respectively to a mean allelic frequency of 0.031. Conversely in the S form, this mutation occurred in a relative higher frequency comparing to the M form (χ^2 ^= 6.75, df = 1, *P *< 0.01) with an equal mean of 0.37 per month.

**Table 3:** Variation in the frequency of the *ace-1*^*R *^mutation and the relative prevalence of the two molecular forms of *Anopheles gambiae *ss in VK7 in September and October 2005

	S form^a^	*ace-1*^*R*b^	F(R)^c^	M form^a^	*ace-1*^*R*b^	F(R)^c^
		
September	11 38%	1RR+6RS+4SS	0.36	18 62%	0RR+1RS+17SS	0.028
October	15 52%	0RR+11RS+4SS	0.37	14 48%	0RR+1RS+13SS	0.036
Total	26 45%	1RR+17RS+8SS	0.37	32 55%	0RR+2RS+30SS	0.031

^a ^Number of mosquitoes analysed, relative prevalence of molecular forms of *An. gambiae *(in percentage)

^b ^Genotype of the *ace-1*^*R *^mutation

^c ^Allelic frequency of the *ace-1*^*R *^mutation in mosquitoes analysed

[the relative prevalence between the M and S forms did not differ significantly during the two months, χ^2 ^= 1.06, df = 1, P > 0.05].

One individual from the S form was detected as a homozygous resistant for this mutation in September. In the S form, all specimens heterozygous for the *ace-1*^*R *^mutation (17 females) had also the *kdr *mutation in heterozygous status. The one individual homozygous *ace-1*^*R*^/*ace-1*^*R *^was also homozygous *kdr*^*r*^*/kdr*^*r*^. Inversely in the M form, the two individual detected as heterozygous *ace-1*^*r*^/*ace-1*^*s *^were homozygous *kdr*^*s*^*/kdr*^*s*^.

## Discussion

In Burkina Faso, current chromosomal and molecular forms of *An. gambiae *s.s. are tightly correlated [4,10]. Formerly Robert *et al*. [13] studying the distribution of *An. gambiae *s.s. cytotypes in the rice field area of Vallée du Kou in 1984 showed a predominance of the *Mopti *chromosomal form. With the progress in molecular genetic, this distribution has been updated in 1999 and 2000 by Diabate *et al *[11,12], pointing out the predominance of the M molecular form corresponding to the *Mopti *chromosomal form. However the occurrence of the S molecular form (corresponding to the *Savanna *chromosomal form) has been observed toward the end of the rainy season. The results obtained in 2005 followed the same pattern of distribution, but the overall proportion of the S form has increased further reaching a maximum of 51% *vs*. 24% at the same period in 2000 [12]. Taking to account that both studies were performed in the same place (VK7) and during the same period (from July to November), it appears that the relative frequency of the S form has increased. Future studies to confirm this trend are encouraged.

The increase in the S form could probably be due to human activities creating new habitats for the S population in this area, such as house building using bricks of banco and muddles taken out from the soil. Such activities increase the number of temporary rain-filled breeding sites like pits, ponds and puddles that are favourable to S form development throughout the village.

In general, in West Africa, the S form is not well adapted to rice paddies, whilst the M form develops rather well [24,25]. However, some of the rice paddies used to grow vegetables and some irrigation canals not well managed could constitute during the rainy season, suitable habitats for the S larvae.

The changing in the vector population structure may presumably increase the malaria transmission level. Even though the malaria vector population was dominated by the M form throughout the year, this form had a low sporozoite rate because of the low parity rate observed within its population [14]. During the sympatry period (October), the sporozoite index in the S form was not statistically different to that of the M form, but these are preliminary results that would need to be confirmed on a bigger sample and during different periods of the year.

The main mechanism conferring resistance of *An. gambiae *to pyrethroids in West Africa, the Leu-Phe *kdr *mutation, did not vary in the S form (93%) compared to its frequency in 1999 and 2000 [12], as expected.

The spread of the *kdr *mutation is an ongoing process in the M form as its allelic frequency in 2005 was five fold higher than in 2000. Indeed six years ago, the *kdr *mutation was found occurring only in the S form and some investigations conducted in VK7 during the rainy season 1999 failed to identify this mutation in the M one [11].

This difference persisted because of the strong reproductive barriers between the two forms relevantly pointed out by some authors [4,26,27]. In November 1999, the *kdr *mutation was identified for the first time in the M form at a very low frequency (0.006) with only one heterozygous RS among 161 specimens tested [12]. The following year, the frequency in the M form increased to 0.02 and all individuals *kdr*-positive were only heterozygous (RS). Now, the proportion of homozygous resistant in the M form is increasing which enhances the overall frequency of this mutation.

The high frequency of the *kdr *mutation in the S form is presumably due to the long-term and extensive use of insecticide for cotton crop protection, DDT in the 1960–1970s replaced by pyrethroids in the 1980s [11]. Then, its spreading from the S to the M form through introgression [28] is a recent and ongoing process, limited in savannah environments of West Africa to the place where *An. gambiae *M/*Mopti *and S/*Savanna *forms are found in sympatry at high densities [29].

Cotton crops are located just on the outside of rice fields. Insecticides applied on cotton during the rainy season may drift or be washed to the rice field during heavy rains conferring a selection pressure even for the M form. Moreover, during the last decade some rice producers also started to grow vegetables in the paddies using the same cotton's insecticides.

These agricultural uses of insecticides, mainly pyrethroids, exert the main selective pressure on the mosquito populations because of the quantity applied (e.g. six rounds of treatment each two weeks on cotton crop during the rainy season) and its action on the larval stage on large population of both sex. The domestic use of pyrethroids, through coils and ITNs, is more selective (it acts only on the anthrophilic fraction of biting females) and probably plays a secondary role on resistance selection in this rural area.

The present results showed that the *ace-1*^*R *^mutation is mostly present in the S form and less frequently in the M one. This finding suggests that the *ace-1*^*R *^resistance allele is evolving along the same pathway like the *kdr *mutation in this area. As for *kdr*, it occurred probably prior in the S form and may acquire by the M form through introgressive hybridization [30]. The selection of the *ace-1*^*R *^mutation in the S form could be related to the increasing use of organophosphates in cotton treatment in mixture with pyrethroids since the end of the 1990s. This insecticide resistance management (IRM) strategy was implemented at a large scale in West Africa to manage the pyrethroid resistance of the main cotton pest, *Helicoverpa armigera *[31].

With the exposure of the M populations to insecticide pressure, the *ace-1*^*R *^mutation began to spread within this form. Similarly to the *kdr *mutation, it will probably increases in frequency within the M populations in the coming years. Additional studies are crucial to determine precisely the origin of this gene among the M form and gene flow pattern between the two forms in natural populations. Alternatively the reproductive fitness associated with this mutation in *An. gambiae *both S and M population remains to be evaluated [32].

The reported change in malaria vectors population structure is mainly driven by human activities and will call for modified malaria control strategies. The increasing of the S form proportion and the emergence of the *ace-1*^*R *^mutation concomitantly with the Leu-Phe *kdr *mutation among the same populations of *An. gambiae *s.s. is an atypical ecological pattern in an irrigated rice growing area. With the expansion of agricultural practices such as vegetable growing, the application of pesticides with different mechanisms of action is rising. This may favour the development of multiple resistance mechanisms in *An. gambiae *[33,34], which is a dynamic process that needs to be carefully monitored at the molecular form level and through designed spatial and seasonal surveys.

Further studies are needed to determine: (i) the phenotypic effects, particularly when the two mutations occur concomitantly and taking into account if metabolic-based resistance is present and (ii) the operational impact of both mutations on the efficacy of pyrethroid or organophosphate/carbamate based vector control.

Until recently, several studies conducted in savannah environment of Ivory Coast (West Africa) showed that pyrethroids treated nets still achieved a good control of *kdr *resistant *An. gambiae *either in experimental huts [35] or in field trials [36]. Indeed recent paper from N'Guessan *et al. *in southern Benin [37] indicated that the *kdr *target insensitivity present at high frequency in M/*Forest *population of *An. gambiae *is associated with the decreased efficacy of ITNs and pyrethroid based IRS. Some alternatives to pyrethroids on ITNs are therefore necessary. Preliminary studies using organophosphate and carbamate treated nets in experimental huts have already shown good results in areas of *kdr *resistance [38,39].

The presence of multiple resistance mechanism in *An. gambiae *in south-west Burkina Faso may constitute an obstacle for the future success of malaria control programmes based on ITNs or IRS with pyrethroids or organophosphates/carbamates. The present study should provide useful information for small and large-scale field trials on insecticide efficacy in this study area.

## Competing interests

The authors declare that they have no competing interests.

## Authors' contributions

DKR participated to the study design, undertook the field study, analysed the data and wrote the paper. DA participated to the study design, the data analysis and the manuscript drafting. LD participated to the study design and the samples analysis in the laboratory. OA participated to the field study and the sample collection. NR participated to the study design. OJB is the administrative authority who facilitated the implementation of the study. JMH designed the study, participated to the data analysis and the drafting of the paper. FC participated to the study design and the data analysis. TB participated to the data analysis and interpretation, the drafting and the revision to the paper. All authors read and approved the final manuscript.
